# Does moral commitment predict resistance to corruption? experimental evidence from a bribery game

**DOI:** 10.1371/journal.pone.0262201

**Published:** 2022-01-11

**Authors:** Carmen Tanner, Stefan Linder, Matthias Sohn

**Affiliations:** 1 Department of Banking and Finance, University of Zurich, Zurich, Switzerland; 2 Chair of Economic Psychology and Leadership Ethics, Zeppelin University Friedrichshafen, Friedrichshafen, Germany; 3 Chair of Accounting & Management Control, ESSEC Business School Paris, Cergy-Pontoise Cedex, France; 4 Chair of Management Accounting, European University Viadrina, Frankfurt (Oder), Germany; Universität Heidelberg, GERMANY

## Abstract

Corruption is ubiquitous in practice and has severe negative consequences for organizations and societies at large. Drawing on a laboratory experiment, we propose that individuals high in moral commitment are less likely to engage in corrupt behaviors and prefer foregoing financial benefits. Specifically, we posit that individuals refrain from corruption (i) the more they endorse integrity (incorruptibility) as a protected value and (ii) the higher their level of Honesty-Humility. The results of a two-step experiment largely support our expectations: people who treat compromises to integrity as unacceptable were less willing to accept bribes, and Honesty-Humility decreased bribe-giving. The findings are robust to demographic variables (e.g., age, gender, cultural background) and additional personal characteristics (e.g., risk tolerance, dispositional greed) and have important implications for ongoing theory-building efforts and business practice.

## Introduction

Corruption, mainly understood as the abuse of entrusted power for personal benefit at the expense of others [1], is still experienced as a widespread and persistent problem causing harm at the economic, political, and social levels [2]. Corruption is also a major concern for organizations as violations of international anti-corruption standards can lead to severe legal and financial consequences. Not surprisingly, understanding the determinants of corruption and how to deter organizational corruption is of utmost interest. So far, research has mostly focused on macro-level antecedents of corruption, such as the role of economic, organizational, institutional, or cultural factors (e.g., [3–11]). These studies have greatly enhanced our understanding of aggregated differences in corruption between countries and companies. However, they do not offer insights into why–even within the *same* (organizational) environment–individuals largely differ in their susceptibility to corruption [12, 13]. While some individuals quickly adopt corrupt practices when faced with a corrupt environment in the workplace, others appear to be more resistant to corruption, despite external pressure or prospects of financial benefits [14–17]. This raises an important issue relevant for research and practice alike: Who is more likely to resist corruption and why? Clearly, this is a question that emphasizes the role of individual differences.

Although there is much evidence on individual differences of unethical behavior (such as dishonesty) in individual settings (e.g., [18, 19]), our knowledge about individual differences in collaborative settings, such as bribery (which by definition requires at least two parties working together for attaining benefits), is underexplored [13]. To date, beyond a small stream of work that has examined the role of sociodemographic characteristics of the individuals acting corruptly, notably the individual’s gender, age, education, or cultural background (e.g., [10, 20–22]), only a few scholars have started to pay more attention to psychological characteristics and intrapersonal dynamics of the individual who acts corruptly (e.g, [12, 15, 16, 23]).

For instance, prior studies indicate that individuals are more likely to engage in corruption when they believe that corruption is justifiable [24], believe that others will be likely to act corruptly [22, 25], perceive corruption not as immoral [16], or are prone to use self-serving rationalizations [26]. In line with a social-psychological perspective emphasizing that norms are vital in explaining individual behavior, empirical studies confirmed that personal and social norms are likely to predict whether individuals adopt corrupt practices [12, 23, 27]. According to the economic approach, an individual’s engagement in corruption is motivated by perceived cost and benefits, including an estimation of the probability of being caught and of the severity of sanctions [28–30]. However, empirical findings testing those claims suggest a rejection of a too simplistic rational model (e.g., [31, 32]). Furthermore, some pioneering empirical studies have revealed a negative association between honesty-humility trait and corrupt behavior [15, 16] and a recent study [33] found that individuals with higher levels of Machiavellian traits were more likely to condone corruption. This underscores that individual traits merit a closer look for better understanding individual-level factors for corrupt behavior.

Shedding more light on why people differ in their susceptibility to engage in corruption not only promises to enhance our understanding of why we see within-company variations in corrupt behavior but helps to refine policy recommendations. Essentially, the aim of the present study is to contribute to the understanding of individual characteristics that help explaining resistance to corruption. A theoretical paper, [14] relates to deontic justice theory [34] and proposes that adherence to deontic principles may underlie people’s resistance to socialization into corrupt practices. This is an appealing suggestion that, however, still awaits empirical examinations. We rely on the conceptual framework of moral intelligence [35], which proposes several psychological conditions that are required to put ethical values into practice. In this model, *moral commitment* is pivotal for moral self-regulation and reflects the motivational power and willingness to strive for moral goals. In this research, we attempt to examine the relationship between moral commitment and refusal to offer and/or accepting a bribe. For this purpose, we rely on two similar but not identical proxies of moral commitment: one’s integrity (incorruptibility) as a *protected value* (e.g., [36, 37]) and *Honesty-Humility* trait (e.g., [38]). Both research lines do not only provide us with reliable scales for empirical research, but both characteristics were also found to predict greater resistance to situational influences in ethical decision-making (e.g., [15, 16, 39, 40]). Therefore, they appear to be suitable candidates for examining individual differences in resistance to corruption.

While corruption can take many forms, the focus of the present study is on a specific but pressing type of corruption: *bribery*. As the United Nations Convention Against Corruption points out, bribery is both unethical and illegal. In addition, bribery is a type of collaborative and interdependent behavior, whereby at least two parties are required to cooperate to attain benefits or services–a “briber” (e.g., a firm, a citizen, someone who gives a bribe) and a “bribee” (e.g., a public official, someone who takes the bribe) [41, 42]. Thus, bribery differs fundamentally from studies that have focused on dishonesty or fraud, whereby an individual can benefit at the expense of others through independent unethical acts. Furthermore, in line with the interdependent character of corrupt transactions and to gain more insights into the explanatory power of intrapersonal factors, we distinguish between *offering a bribe* and *accepting or rejecting a bribe*. Corrupt transactions usually involve two sides–individuals who offer, give, or promise money, goods, or services, and individuals who take or accept money or a gift in exchange for the abuse. As several authors criticize (e.g., [12, 43]), remarkably few studies have examined the behavior of both sides. We also believe it is essential to consider both sides of bribery since they may involve different motivations.

Since bribery is a crime and transgressors are therefore more likely to cover up their involvement, observing and assessing corrupt behavior is difficult. Most empirical studies on corrupt behavior are based on self-reports. Surveys, however, measure intentions rather than actual behaviors. We decided to take a (quasi) experimental approach to be able to observe corrupt behavior credibly in a controlled environment.

Our study draws on data from a two-part experiment. In the first step, we administered an online survey to assess demographic information and various personal values and characteristics. Approximately three weeks later, these participants took part in a fully incentivized bribery game (adopted from [10]). For this purpose, participants were randomly assigned to the role of briber (“private citizen”), bribee (“public official”), or member of a group of individuals who are harmed by corruption (“other member of society”). Participants were matched, and the “private citizen” could offer the “public official” a bribe, and the “public officials” could accept or reject bribes. A successful bribe financially benefits the briber-bribee pair and reduces the remuneration of the respective “other member of society” participant. This design choice allowed us to test the strength of the two moral commitment variables to predict both offering *and* accepting a bribe.

We make several contributions to existing corruption research. First, we contribute to the sparse empirical literature on who may be more likely to resist corrupt acts. In doing so, we take a methodological approach that allows us to observe corrupt behavior rather than to build on self-reported behavior. This is particularly important given the well-known discrepancy between individual’s values (or attitudes) and their behavior. It is a major issue in many real-life domains that that what people say or claim to value does not necessarily correlate with what they do (e.g., [44–47]). Although survey-based corruption research has also its advantages, assessing corrupt behaviors by self-reports implies a risk of social desirability bias. Through a laboratory experiment, we get a better understanding of which individual variables are more powerful in becoming translated into action and hence contribute to prior experimental research on corruption that has gained popularity in recent years (e.g., [48]).

Second, we make theoretical contributions. According to [14], individuals may be resistant to corruption because of their adherence to deontological principles. Likewise, the theoretical framework of moral commitment considers that people view moral values as obligations, which manifests itself in a strong desire and willingness to accordance with those values. In this study, we use protected values and Honesty-Humility as two expressions of moral commitment. As will be outlined below, however, both constructs suggest different reasons for resistance. Hence, we add to a better understanding of why some individuals refrain from corrupt acts, despite the prospect of personal gains.

Finally, the present research has implications for practice. As some authors have pointed out [12], since organizational cultures are generally more difficult to change, accounting for individual differences might be more effective. Indeed, the fact that much corruption remains *hidden* highlights the importance of complementing or replacing costly monitoring and control strategies, e.g., by strategies that support strengthening moral values or help selecting or promoting employees to managerial positions based on moral characteristics.

### Moral commitment and corruption

The present study contends that individuals are more likely to abstain from engaging in corrupt transactions when they are intrinsically committed to moral principles. Moral commitment is defined as the strong desire and willingness to strive consistently and persistently for desirable goals [35]. In line with arguments of other moral self-regulatory perspectives (e.g., [34, 49, 50]), moral commitment is associated with the following main characteristics and implications for corrupt behavior.

First, moral commitment reflects a steadfast adherence to moral values. It is often seen as being associated with a deontological perspective. Unlike the consequentialist perspective, a deontological principle refers to an “ought” that is considered as an end in itself rather than a means [34, 51–53]. Since moral commitment produces a heightened sense of obligation to behave consistently with those principles, it serves as a robust motivational driver for self-regulatory processes directed to act according to one’s moral standards [14, 50]. Moreover, moral commitment is closely connected to individuals” sense of themselves as having personal integrity, as being authentically moral, consistent with one’s values and convictions [54]. Second, moral commitment links the self to moral principles. It reflects strong moral stances and core beliefs central to one’s identity [55, 56]. Thus, compromising one’s moral standards could threaten one’s sense of personal and public identity [57, 58]. Finally, moral commitment is associated with intrinsic moral costs. Since morality is part of one’s self-understanding, behaving in ways that violate one’s moral standards can generate negative feelings, such as guilt or shame [49, 56, 59]. According to [49], the anticipation of internal sanctions can also powerfully shape behavior. Because moral commitment is accompanied by a strong urge to comply with moral values to avoid threats of one’s private and public moral identity, morally committed individuals are endowed with motivational strength to defend integrity (i.e., incorruptibility), and they are likely to do so by resisting corruption.

To empirically investigate the relationship between moral commitment and corrupt behavior, we rely in the present study on two well-established moral conceptualizations which are related to moral commitment: protected values and the Honesty-Humility trait. We focus on these two approaches since both characteristics have been identified in prior research to set boundaries to the influence of situational factors (such as controls, sanctions, financial incentives) in ethical decision-making (e.g., [39, 40]). Thus, it seems reasonable to contend that both variables are suitable candidates to predict resistance to both offering and accepting or rejecting a bribe.

### Protected values

Protected values represent a particular type of value within an individual’s set of values. These values are entities or behavioral standards that people believe ought to be absolute and protected from (utilitarian) trade-offs because they tap into ethical principles and are central to people’s moral identity. Such values are conceived as non-compensatory, incommensurable, and “not for sale” [36, 60–62].

This notion is well in line with findings suggesting that protected values are often rooted in non-consequentialist, deontological principles that imply morally mandated actions or prohibitions to protect those standards [36, 61]. Also consistent with the above-mentioned characteristics of moral commitment, experiencing or witnessing that protected values may be compromised is threatening and therefore likely to trigger reactions of blameworthiness and outrage [58, 60]. When witnessing violations of protected values by others, this can even induce sanction intentions [63]. Interestingly, studies have shown that people endorsing protected values are more resistant to situational influences, such as financial incentives. For instance, studies found that people treating honesty as a protected value were less likely to compromise honesty for financial gains [39]. Indeed, previous research found protected values to be the strongest predictor of resistance to economic incentives associated with cheating [64]. People endorsing protected values were also found to be less sensitive to framing effects (i.e., framing consequences as losses or gains) [36].

The most current measure of protected values highlights the importance of separating two forms of individual stances when such values are “put at risk” [37, 39]. The cognitive component reflects an individual’s position that a particular moral or behavioral standard (such as integrity, incorruptibility) is non-tradable (PV_NT_ = no trade-offs with protected values). The affective component refers to *reactions of blameworthiness* to violations of moral or behavioral standards (PV_RC_ = reactions to compromising protected values), threatening both people’s private and public identity.

In our context, we content the behavioral standard being treated as a protected value and being seen as essential for building resistance to corruption is *integrity*. Although integrity has several meanings (for a review of the definitions of integrity, see [65]), we intend for integrity to mean being consistent with one’s internalized moral values. In other words: being *incorruptible*. The logic underlying the perspective of protected values is essentially this: some people may view bribing or accepting bribes as putting their “integrity”, their sense of being incorruptible at risk. To the extent that people deem integrity as non-tradable and compromising integrity as unacceptable, they should protect it by refusing corruption. We believe that both components of protected values reflect a strong adherence to incorruptibility that is even strong enough to overcome the value action gap. This leads us to our first hypothesis:

Hypothesis 1: The more individuals endorse integrity (incorruptibility) as a protected value, the less they engage in offering or accepting bribes.

### Honesty-Humility

The second construct reflecting one’s moral commitment is *Honesty-Humility*. Honesty-Humility is one of the six personality dimensions of the HEXACO personality model (the successor model of the Big Five model of personality). Honesty-Humility is understood as a broad dimension that includes personal qualities, such as sincerity, fairness, greed avoidance, and modesty [38]. Honesty-Humility is defined as “the tendency to be fair and genuine in dealing with others” ([66] p. 156). It reflects “people’s willingness to refrain from exploiting others or bending the rules and norms–even if such actions would be individually beneficial and bear little risk of retaliation or sanctions” ([19] p. 73).

High levels of Honesty-Humility are associated with less nonexploitation [67], including counterproductive work behavior and workplace delinquency [68], more altruism [69], and more cooperative behavior [40]. Honesty-Humility has also been shown to play a crucial role in ethical decision-making. Across six studies with different cheating tasks, incentive structures, and samples, [19] found that Honesty-Humility was negatively related to cheating. Additionally, a negative relationship between Honesty-Humility and dishonesty has been observed in a series of studies (e.g., [70, 71]). In a large-scale re-analysis, researchers concluded that the negative relationship between Honesty-Humility and dishonesty had a medium-to-large effect size [18]. Especially important for this current research, [15, 16] found that people with higher scores on Honesty-Humility were less likely to cheat in situations allowing for corrupted collaboration, and less likely to take a bribe. Finally, studies have demonstrated that people high in Honesty-Humility are also less likely to condition their behavior on situational cues, they remain relatively stable in honesty across different situational manipulations (such as different sizes of financial incentives, or moral and immoral primes) [40, 72, 73].

Hence, we posit that Honesty-Humility is related to people viewing bribing or accepting bribes as being inconsistent with their disposition of being fair and genuine in dealing with others, thereby increasing their resistance to corruption. We hypothesize:

Hypothesis 2: The higher their level of Honesty-Humility, the less individuals engage in offering or accepting bribes.

## Method and materials

To test our predictions, we draw on a two-stage cross-subject laboratory experiment with students enrolled in various programs at a major French metropolitan business school. The study was approved by the University’s ethics committee and was conducted in accordance with the Declaration of Helsinki convention and its later amendments. Written consent was obtained by participants first registering explicitly for taking part in an experiment in the lab and by responding at the beginning of the actual experiment to a screen informing them about the experiment, the possibility to withdraw at any time, and asking them to click on a button if they wish to start the study. The experimental instructions and the survey items are reproduced in S1 Appendix. S2 Appendix offers a flow chart of the study.

In the first stage (time t_0_), an online survey was used to gather data on participants’ characteristics, notably their protected value for integrity, Honesty-Humility, and a set of additional psychological characteristics (e.g., risk propensity, greed) and demographic variables to mask the moral content. Thus, all independent and control variables were polled at t_0_. To ensure anonymity, participants were asked to generate their own personal identification code, which they needed again for the experiment in the computer lab. The code is composed of the first two letters of the town in which they were born, the first two letters of the first name of their mother, and the day of their birthday. Three weeks later (at time t_1_), the participants played the 2005 version of the fully anonymous one-shot bribery game initially developed by [10]. To minimize interactions between participants and the experimenter, the experiment was computerized using z-Tree software [74]. Our two-part study allows us to poll all independent variables prior to the laboratory experiment with a sufficiently long-time lag. By collecting participants’ protected value for integrity and Honesty-Humility in t_0_ and by not giving participants any information at t_0_ about the content of the laboratory part at t_1_, our design rules out potential order effects and reduces demand effects.

In each session of 15 or 18 participants, each participant was assigned to one of three roles: the role of a “private citizen” (Player 1), a “public official” (Player 2), or “another member of society” (Player 3). We used terms such as “private citizen” instead of Player 1 and “bribe” instead of transfer. Prior research shows that such framing effects have little effect in corruption games [75]. The “private citizen” can offer a bribe to the “public official,” and when the bribe is paid and accepted, the briber-bribee pair benefits, but the “other member of society” suffers a monetary loss. As an incentive to participate in the study, all participants received a show-up fee of 10 EUR and an additional maximum of 16 EUR depending on their and others’ decisions. The participants played with the currency “Gilpets” (G), with an exchange rate of 3 G for 1 EUR. In line with [10], the initial endowments were 35 G for the “private citizen,” 35 G for the “public official,” and 25 G for the “other member of society.”

The remuneration scheme mirrored prior research [10]. Each “private citizen” receives an initial endowment *Y*_*c*_ 35 G and is able to offer a bribe, *b* (between 1 G and 20 G), in exchange for a corrupt service provided by the “public official” with the value *V* (16 G). Every “private citizen” offering a bribe incurs a cost, *E*, (1 G) representing the risk of being caught and punished. The initial endowment of each “public official” equals *Y*_*p*_ (35 G). If she accepts an offered bribe, she must provide the corrupt service and incurs a cost, *K* (5 G), which is similar to the cost of being caught and punished, the cost of supplying the corrupt service, and the cost of any effort undertaken to reduce the risk of being caught. The remaining participants, “other members of society,” receive the initial endowment *Y*_*o*_ and, unlike “private citizens” and “public officials,” are unable to make their own decisions to influence their payoff. Instead, for every bribe offered by a “private citizen” and accepted by a “public official,” each “other member of society” incurs a cost, *h*. Therefore, the final payoff of the “private citizen” is *F*_*c*_ = *Y*_*c*_ if she chooses not to offer a bribe, *F*_*c*_ = *Y*_*c*_−*E*+*V*−*b* if she offers a bribe that is accepted, and *F*_*c*_ = *Y*_*c*_−*E* if she offers a bribe that is rejected. The final payoff for any “public official” who does not accept a bribe remains *F*_*p*_ = *Y*_*p*_. “Public officials” who accept a bribe receive *F*_*p*_ = *Y*_*p*_−*K*+*b*. The “other members of society” receive a final payoff equal to *F*_*o*_ = *Y*_*o*_−*N*_*c*_*h*, where *N*_*c*_∈{1, 2, 3, 4, 5, 6} is the number of pairings of “private citizens” and “public officials” within the session who offer and accept a bribe. The remuneration scheme is designed such that there is an incentive to bribe or accept bribes. Specifically, self-maximizing “public officials” will accept any bribe that is greater than the costs associated with accepting the bribe (*b*>*K*), and each “private citizen” will offer bribes of *K*+*p*, where *p* is a small positive amount (given that *K* was set to 5 G in our setting, the subgame perfect equilibrium bribe equals 6 G). After completing the experiment, the participants’ payoffs were calculated in z-Tree [74] and distributed in a separate room by a person different from the experimenter.

Participants in the “private citizen” role were first asked whether they want to offer a bribe and, if they agreed, asked how much they would offer the “public official.” We used the strategy method for participants in the “public official” role. For each bribe, *b* (between 1 G and 20 G), each “public official” was separately asked whether she would be willing to accept the bribe. For each “private citizen”–“public official” pairing, this allowed us to determine whether the bribe was successful and to ultimately calculate the final payoff (all of which was done via z-Tree).

Thus, combining the data on the respondents’ characteristics with their behavior in the bribery game allows us to test the role of individual characteristics, which were collected at t_0_, in predicting corrupt behaviors at t_1_. Thus, the two-stage nature of the experiment allows us to directly measure the individual characteristics that explain why some individuals engage in corrupt behaviors, but other individuals do not bribe or accept bribes.

### Participants

A total of 225 business students participated in this study. The students had different cultural backgrounds (only 37% were French), allowing us to meaningfully control for cultural differences as a predictor of corrupt behaviors. Of all participants, 45% were male, and their median age was 23 years (*SD* = 2.31, range: 18–36 years). The participants were randomly allocated to three roles: one-third of them played the game in the role of a “public official,” one-third played the role of a “private citizen,” and the remaining third played the role of “other members of society.” The participants playing the roles of “private citizen,” or “public official” could engage in corrupt behaviors by either offering or accepting a bribe; however, the “other members of society” could not. These participants did not make decisions; instead, they simply served as real individuals being affected by the decisions. Thus, they served the sole purpose of ensuring that participants taking over the roles of “private citizens” and/or “public officials” affected real individuals with their decisions and not a fictitious person or a computer. Suppose only a fictitious person or computer was affected. In that case, the participants playing the roles of “public official,” or “private citizen” may behave differently from how they would behave in a “real” setting.

Thus, the design by [10] offers greater ecological validity than a game with only the computer playing “other members of society.” However, the design implies that one-third of the participants do not provide us with relevant data; as a consequence of the lack of behavioral observations from the “other members of society,” the effective sample for testing our hypotheses is smaller than the total number of participants (*N* = 152). Of these 152 participants, 46% were male (vs. 45% for the full sample), and the median age of 23 years (*SD* = 2.38) remains unchanged. The participants assigned to the role of either the “private citizen” or the “public official” do not differ according to age, gender, or the number of semesters enrolled, suggesting that the randomization was successful. On a scale from 0 (basic English language knowledge) to 100 (completely like a native speaker), on average, the participants rated their skills at 79.5, implying that they have advanced English language skills (the instructions were given in English).

### Measures

We used established instruments to assess a participant’s moral commitment.

#### Protected values

This measure consists of two subscales. These two distinct but related subscales of protected values were developed and tested by [37] (see also [39]). PV_RC_ (= reactions to compromising protected values), is designed to capture reactions of blameworthiness to potential violations of ethical principles. To assess PV_RC_, the participants were first presented with two different morally questionable situations; each of the two scenarios described a situation in which a bribe was offered at the workplace. Using a 5-point scale, the participants were then asked–for both scenarios–the extent to which *proposing* (and *accepting)* such an offer was from their perspective (1) *not at all praiseworthy-very praiseworthy*, (2) *not at all blameworthy-very blameworthy*, (3) *not at all outrageous-very outrageous*, and (4) *not at all acceptable-very acceptable* (resulting in a 16-item scale, *α* = 0.87).

The other subscale, PV_NT_ (= no trade-offs with protected values) is designed to capture the extent to which individuals consider integrity as something that should not be traded off for other (financial) entities. To assess PV_NT_, the participants were asked to rate the extent of their agreement with four statements: “Integrity is a value…” (1) “that one should not sacrifice, no matter the benefits,” (2) “for which I think it is right to perform a cost-benefit analysis,” (3) “that cannot be measured in monetary terms,” and (4) “about which I can be flexible if the situation demands it” *(α* = 0.71); the items were rated on a scale from 1 (*strongly disagree*) to 5 (*strongly agree*). Each subscale was calculated as the average score across the corresponding items, with higher scores indicating higher levels of PV_RC_ and PV_NT_, respectively.

#### Honesty-Humility

Individual scores on this scale were based on 10 items encompassing the Honesty-Humility dimension from the HEXACO personality model [38]. The items were designed to assess personal qualities such as sincerity, fairness, greed avoidance, and modesty [38]. All items employed a 5-point response scale (1 = *strongly disagree*; 5 = *strongly agree*) (*α* = 0.66). Sample items are: “I wouldn’t pretend to like someone just to get that person to do favors for me” and “I would get a lot of pleasure from owning expensive luxury goods.” The items were averaged to create an index of Honesty-Humility. Higher scores indicate higher levels of Honesty-Humility.

#### Controls

To facilitate teasing out the predictive strength of the two characteristics of interest and to avoid confounding effects, we collected a set of additional measures. In addition to the demographic variables such as age, we asked for participants’ current semester. Accounting for semesters might be informative, as with increasing number of semesters, participants studying business and/or economics may become more skilled at strategic thinking and/or willing to maximize their remuneration in economic experiments. We also included the following trait-related variables: Trait Competitiveness ([76], 4 items, *α* = 0.82; sample item “I enjoy working in situations involving competition with others”), Dispositional Greed ([77], 6 items, *α* = 0.79; sample item “One can never have enough”), and Community Commitment ([78], 8 items, *α* = 0.82; sample item “I feel that it is important to serve as a volunteer in my community”). All variables were assessed on a scale ranging from 1 (*strongly disagree*) to 7 (*strongly agree*). Averaging across the corresponding items, higher scores indicate higher levels of those traits. We also measured participant’s Risk Tolerance [79] on single item scale from 1 (*not at all willing to take risks)* to 10 (*very willing to take risks*), and whether they would describe themselves as religious. Religiosity was assessed using two items (*α* = 0.81), reflecting how religious participants describe themselves (1 = *not at all religious*; 5 = *very religious*).

Finally, similar to [10], we included a cross-cultural measure of corruption drawing on the Corruption Perception Index (CPI) by Transparency International, in order to include some information on participants’ cultural backgrounds. Though this perception-based measure has been criticized on methodological grounds, we chose this measure because it is one of the most widely used indicators of inter-cultural differences in corruption [2, 80]. We first asked participants, whether they are French and, if they disagreed, asked about the country they culturally identify with. Based on the CPI the participants’ cultural backgrounds were clustered into 3 groups. As indicated by the CPI, countries with low perceived corruption take the value of 1, countries with medium perceived corruption take the value of 2, and countries with high perceived corruption take the value of 3. This clustering allows us to control for the potential role of differences in cultural identities in predicting participants’ propensity to engage in corrupt behaviors.

## Results

### Descriptive statistics

We focus on a participant’s decision to either engage in or abstain from corrupt behavior. Analogous to [10], our main results are not based on the size of the bribe because the size of a bribe is a strategic decision (see the Robustness Section for details). Specifically, the dependent variable “Bribe-Offering” takes the value of 1 if a “private citizen” decides to offer a bribe and 0 if she chooses not to offer a bribe regardless of the amount she chooses to offer. Likewise, the dependent variable “Bribe-Accepting” takes the value of 1 if a “public official” decides to accept any offer (between 1 G and 20 G) and 0 if she chooses not to accept an offer. “BribeTotal" is a dichotomous dummy for which 1 represents either giving or accepting a bribe and 0 otherwise. Of the 76 participants assigned to the “private citizen” role, 60 (79%) chose to offer some positive amount, whereas the remaining 16 (21%) chose not to offer a bribe. Of the 76 participants who were assigned to the role of “public official,” 63 (83%) decided to accept a bribe, whereas 13 (17%) “public officials” chose not to accept any offers. For the full sample, this results in 123 (81%) “private citizens” and “public officials” either offering or accepting a bribe. Given the high number of participants engaging in corruption, participants in the “private citizens” role earned an average variable remuneration of 12.24€, “public officials” earned on average 12.23€, and “other members of society” earned on average 5.54€ in variable remuneration (in addition to the 10€ show-up compensation for all participants).

Table 1 shows the descriptive statistics for all variables of interest, and Table 2 presents the pairwise correlations among these variables.

**Table 1:** Summary statistics.

Variable	Obs	Mean	Std.Dev.	Min	Max
Bribe-Offering	76	0.79	0.41	0.00	1.00
Bribe-Accepting	76	0.83	0.38	0.00	1.00
BribeTotal	152	0.81	0.39	0.00	1.00
PVnt	152	3.68	0.93	1.25	5.00
PVrc	152	4.11	0.63	2.06	5.00
Honesty-Humility	152	4.39	0.98	2.10	7.00
Dispositional Greed	152	4.60	1.21	1.00	7.00
Community Commitment	152	4.63	1.16	1.67	7.00
Trait Competitiveness	152	5.06	1.29	1.00	7.00
Risk Tolerance	152	6.51	2.34	1.00	10.00
CPI	152	1.55	0.65	1.00	3.00
Age	152	22.95	2.38	18.00	36.00
Gender	152	0.54	0.50	0.00	1.00
Semesters	152	1.63	1.13	1.00	5.00
Religiosity	152	2.46	1.33	1.00	5.00

*Note*. Bribe-Offering is coded as 1 = *offering a bribe*, 0 = *not offering a bribe*. Bribe-Accepting is coded as 1 = *accepting*, 0 = *not accepting a bribe*. BribeTotal is a combined variable of Bribe-Offering and Bribe-Accepting and is 1 when a participant chooses to offer or accept a bribe, and 0 otherwise. Higher moral commitment scores indicate greater endorsement of PVnt, PVrc and Honesty-Humility. Higher trait-related scores indicate higher levels on Dispositional Greed, Community Commitment and Trait Competitiveness. Higher scores on Risk Tolerance indicate higher willingness to take risks. Gender is dummy coded as 1 = *female*, 0 = *male*. Religiosity reflects whether participants describe themselves as 1 (*not at all religious*) to 5 (*very religious*). CPI is coded as 1 = *low perceived corruption*, 2 = *medium perceived corruption*, 3 = *high perceived corruption*. Semesters measures in what year of the program the participants are currently in.

**Table 2:** Pairwise Pearson’s correlations.

Variables	(1)	(2)	(3)	(4)	(5)	(6)	(7)	(8)	(9)	(10)	(11)	(12)	(13)	(14)	(15)
(1) Bribe-Offering	1.00														
(2) Bribe-Accepting		1.00													
(3) BribeTotal	1.00***	1.00***	1.00												
(4) PVnt	-0.04	-0.03	-0.04	1.00											
(5) PVrc	-0.11	-0.21*	-0.17**	0.39***	1.00										
(6) Honesty-Humility	-0.12	-0.19*	-0.16*	0.50***	0.34***	1.00									
(7) Disp. Greed	-0.05	0.30***	0.12	-0.25***	-0.21***	-0.37***	1.00								
(8) Com. Commitment	-0.12	-0.02	-0.06	0.10	0.16*	0.10	-0.08	1.00							
(9) Trait Competitiveness	-0.01	0.17	0.07	-0.29***	-0.13*	-0.38***	0.36***	0.04	1.00						
(10) Risk Tolerance	-0.01	0.05	0.02	-0.12	-0.14*	-0.09	0.25***	0.13	0.24***	1.00					
(11) Age	-0.04	-0.06	-0.04	0.14*	0.07	0.08	0.04	-0.08	-0.17**	0.09	1.00				
(12) Gender	0.00	-0.09	-0.05	0.21**	0.17**	0.21**	-0.20**	-0.03	-0.28***	-0.20**	0.09	1.00			
(13) Religiosity	-0.18	0.19*	-0.01	0.15*	0.07	0.00	0.04	0.11	0.06	-0.04	0.03	-0.01	1.00		
(14) CPI	-0.01	0.00	0.00	0.02	0.02	-0.01	0.05	0.22***	-0.08	-0.02	0.18**	0.12	0.21***	1.00	
(15) Semesters	0.19*	-0.13	0.03	0.10	0.09	0.10	-0.08	-0.04	0.03	-0.15*	0.21***	-0.11	-0.05	0.04	1.00

*Note*. Gender is dummy coded as 1 = *female*, 0 = *male*. CPI is coded as 1 = *low perceived corruption*, 2 = *medium perceived corruption*, 3 = *high perceived corruption*. *N* (BribeTotal) = 152; *n* (Bribe-Offering) = 76; *n* (Bribe-Accepting) = 76.

* *p* < .10

** *p* < .05

*** *p* < .01.

The pairwise correlations presented in Table 2 suggest that PV_RC_ and Honesty-Humility are negatively related to corrupt behavior (BribeTotal). PV_NT_ does not correlate with giving or accepting a bribe. Dispositional Greed is positively correlated with accepting a bribe. We also observe that the variables that measure different facets of participants’ moral values correlate. Honesty-Humility correlates significantly with PV_RC_ and PV_NT_, which is not surprising given that both constructs represent different facets of one’s moral commitment. Competitiveness and Dispositional Greed correlate negatively with PV_RC_, PV_NT_ and Honesty-Humility. This is what one would expect given that individuals high in protected values are less susceptible to financial incentives to engage in unethical behavior and Honesty-Humility entails greed-avoidance as a core facet. Importantly, tests to see if the data met the assumption of collinearity indicated that multicollinearity was not a concern. The variance inflation factor (VIF) is below 2.0 and Tolerance is greater than .6 for all variables displayed in Table 2. Consequently, we proceed to the multivariate analyses

### Multivariate analyses

Table 3 presents the results of seven regressions. The first five models relate to the pooled sample of both “private citizens” and “public officials” (*N* = 152). The sixth model shows the results only for “private citizens,” whereas the seventh model shows the results for the participants in the “public official” role (both *n’*s = 76). Because the dependent variable in all models is binary (engaging or abstaining from bribing and accepting or rejecting a bribe), we calculate probit regressions. In addition to the explanatory variables of interest, all probit models include the participants’ age and gender [22], their religiosity, and current semester as control variables. We also control for CPI because [10] found that students’ cultural background predicts engagement in corrupt behavior.

**Table 3:** Probit analyses of engagement in bribery.

	(1)	(2)	(3)	(4)	(5)	(6)	(7)
VARIABLES	BribeTotal	BribeTotal	BribeTotal	BribeTotal	BribeTotal	Bribe-Offering	Bribe-Accepting
PVrc		-0.452** (0.182)	-0.492** (0.213)		-0.429** (0.199)	-0.143 (0.316)	-0.679** (0.289)
PVnt			0.071 (0.143)		0.188 (0.145)	0.146 (0.210)	0.283 (0.179)
Honesty-Humility				-0.262* (0.138)	-0.265** (0.118)	-0.295* (0.160)	-0.256 (0.249)
CPI	0.037 (0.172)	0.008 (0.187)	0.011 (0.186)	0.035 (0.174)	0.023 (0.183)	0.128 (0.344)	-0.169 (0.279)
Age	-0.032 (0.061)	-0.021 (0.056)	-0.024 (0.057)	-0.027 (0.057)	-0.027 (0.056)	-0.032 (0.067)	0.018 (0.093)
Gender	-0.111 (0.204)	-0.019 (0.193)	-0.045 (0.195)	0.011 (0.182)	0.006 (0.188)	0.209 (0.312)	-0.142 (0.276)
Semesters	0.053 (0.085)	0.088 (0.091)	0.080 (0.085)	0.096 (0.100)	0.103 (0.097)	0.468*** (0.141)	-0.079 (0.162)
Religiosity	-0.009 (0.129)	0.009 (0.121)	0.003 (0.123)	-0.005 (0.130)	-0.008 (0.123)	-0.188 (0.152)	0.209 (0.130)
*N*	152	152	152	152	152	76	76
McFadden *Pseudo R*^*2*^	0.006	0.037	0.038	0.032	0.058	0.110	0.134

*Note*. BribeTotal is 1 when a participant chooses to offer or accept a bribe, and 0 otherwise. Bribe-Offering, the dependent variable in column (6), represents the “private citizens” only, and is coded as 1 = *offering a bribe*, 0 = *not offering a bribe*. Bribe-Accepting is the dependent variable in column (7), represents the “public officials” only, and is coded as 1 = *accepting*, 0 = *not accepting a bribe*. Gender is dummy coded as 1 = *female*, 0 = *male*. CPI is coded as 1 = *low perceived corruption*, 2 = *medium perceived corruption*, 3 = *high perceived corruption*. Robust standard errors are reported in parentheses.

* *p* < .10

** *p* < .05

*** *p* < .01.

However, the results in column (1) show that like the other control variables, culture is not related to corrupt behavior in our sample. In column (2), we include the affective subscale of protected values, PV_RC_, in the regression, and the model suggests that PV_RC_ is negatively related to bribing. The direct subscale of protected values measuring the cognitive notion of protected values, PV_NT_, is added in column (3); PV_NT_ is not related to bribing. Thus, our results lend partial support to our hypothesis 1. That is, for PV_RC_, we find that individuals who show reactions of blameworthiness to observed violations of ethical principles are more likely to abstain from bribing and forego monetary benefits. In turn, the non-significant coefficient of PV_NT_ shows that treating integrity as a non-tradable value is not an issue in predicting bribing behavior in our setting. We next calculate a model that includes the control variables and Honesty-Humility without the protected values subscales. Column (4) shows, as proposed, that Honesty-Humility is negatively related with bribing, supporting hypothesis 2. We propose protected values and Honesty-Humility to be closely related but distinct facets of moral commitment. Accordingly, we expect both to predict corrupt behavior with and without controlling for the other construct. We run a final model for the full data where the protected values subscales as well as Honesty-Humility are included into a single regression. The results in column (5) indeed show that both PV_RC_ and Honesty-Humility remain negative and significant. Taken together these results support our hypotheses that protected values and Honesty-Humility are important traits predicting corrupt behavior.

Next, we turn to the separate analyses of bribers and bribees; these analyses provide a more nuanced evaluation of how moral commitment is associated with offering or accepting a bribe. The regression in column (6) shows the results for participants in the “private citizen” role. The dependent variable, Bribe-Offering, takes the value of 1 if the briber offers any bribe and 0 if the briber does not offer a bribe. For participants in the briber role, we observe that Honesty-Humility remains negatively and significantly related to Bribe-Offering. PV_RC_ is not related to Bribe-Offering. However, the picture reverses for Bribe-Accepting in column (7). In the regression, we include participants in the “public official” role, and the dependent variable Bribe-Accepting takes the value of 1 if the “public official” accepts a bribe and 0 otherwise. The model shows that the more participants endorse PV_RC_, the less they accept bribes, whereas Honesty-Humility does not appear to be related to accepting bribes. Taken together, these results suggest that the predictive strength of Honesty-Humility in the full sample is essentially based on the behavior of bribers, while the predictive strength of PV_RC_ is based on the behavior of bribees.

### Robustness and further analyses

To test the robustness of our findings and rule out confounding effects, we also tested whether the effects of PV_RC_ and Honesty-Humility hold when controlling for additional personal characteristics, such as Dispositional Greed, Trait Competitiveness, Community Commitment, and Risk Tolerance.

Considering Dispositional Greed seems important since in much of the public debate about unethical behavior, greed is pinpointed as an underlying reason for such behaviors. Furthermore, to some degree, the Honesty-Humility scale already covers greed avoidance [38]. Honesty-Humility has a broad dimension that includes personal qualities, such as sincerity, fairness, modesty, and greed avoidance [38]. Hence, we draw on the inverse of greed avoidance: an individual’s Dispositional Greed. Dispositional greed is defined as an individual’s insatiability and excessive striving for more goods and money at the expense of others [77, 81].

In a similar vein, Trait Competitiveness has been associated with unethical behaviors (e.g., [82]). Competitiveness may be positively related to corruption if, as is the case in our setting, offering or accepting a bribe can place the individual in a financially superior position relative to her peers. In contrast, Community Commitment implies that an individual places value on developing and maintaining good relational ties with the local community [83]. Because engaging in corrupt behaviors in our experiment reduces the payoffs to the “other members of society,” individuals high in Community Commitment might be less likely to engage in corrupt behaviors than those low in such commitment. Finally, in prior research, individuals with higher Risk Tolerance have been found to be more inclined to engage in unethical behaviors (e.g., [84]). Hence, our results related to PV_RC_ and Honesty-Humility might be confounded with Risk Tolerance.

We ran three additional models, including the moral commitment proxies previously tested (PV_RC_, PV_NT_, and Honesty-Humility), the other personal characteristics, and the demographic variables (see Table 4). Model (1) shows the results for the full sample, and Models (2) and (3) separately show the results for Bribe-Offering and Bribe-Accepting. We generally find that the results of PV_RC_ and Honesty-Humility hold when controlling for additional personal characteristics.

**Table 4:** Robustness analyses of engagement in bribery.

	(1)	(2)	(3)
VARIABLES	BribeTotal	Bribe-Offering	Bribe-Accepting
PVrc	-0.401* (0.214)	-0.028 (0.330)	-0.663** (0.292)
PVnt	0.198 (0.131)	0.201 (0.218)	0.281 (0.196)
Honesty-Humility	-0.236* (0.129)	-0.425** (0.166)	-0.119 (0.312)
Disp. Greed	0.099 (0.129)	-0.172 (0.161)	0.277* (0.145)
Trait Competitiveness	-0.010 (0.104)	0.028 (0.141)	0.094 (0.254)
Com. Commitment	-0.056 (0.108)	-0.267** (0.125)	0.039 (0.125)
Risk Tolerance	0.003 (0.068)	0.051 (0.109)	-0.004 (0.076)
CPI	0.048 (0.195)	0.296 (0.393)	-0.204 (0.336)
Age	-0.039 (0.046)	-0.046 (0.062)	0.014 (0.096)
Gender	0.016 (0.239)	0.161 (0.432)	-0.030 (0.410)
Semesters	0.121 (0.084)	0.516*** (0.181)	-0.086 (0.155)
Religiosity	-0.010 (0.129)	-0.244 (0.150)	0.189 (0.160)
*N*	152	76	76
McFadden *Pseudo R*^*2*^	0.065	0.148	0.185

*Note*. BribeTotal is 1 when a participant chooses to offer or accept a bribe, and 0 otherwise. Bribe-Offering, the dependent variable in column (6), represents the “private citizens” only, and is coded as 1 = *offering a bribe*, 0 = *not offering a bribe*. Bribe-Accepting is the dependent variable in column (7), represents the “public officials” only, and is coded as 1 = *accepting*, 0 = *not accepting a bribe*. Gender is dummy coded as 1 = *female*, 0 = *male*. CPI is coded as 1 = *low perceived corruption*, 2 = *medium perceived corruption*, 3 = *high perceived corruption*. Robust standard errors are reported in parentheses.

* *p* < .10

** *p* < .05

*** *p* < .01.

Additionally, we observe a positive and marginally significant relationship between Dispositional Greed and the participants’ willingness to accept bribes. This result is consistent with evidence recently provided by [85], who found both in a laboratory setting and within a representative sample of the United States, Belgium, and Dutch populations that greedy individuals exhibit less moral behavior. That our results for the role of greed are in line with their findings suggests that our data are suitable for finding relationships related to (im)moral behavior and that our findings are not simply an idiosyncratic consequence of the use of a convenience sample of students or data collection via a laboratory study.

The results also show that Community Commitment is negatively related to accepting bribes. This finding is consistent with the idea that being committed to the community implies that individuals value developing and maintaining good relational ties with their local community [83]. Accepting a bribe might hurt such ties. Other personality variables are unrelated to corrupt behavior. Most importantly the results of our main variables of interest seem robust when controlling for the additional variables. This lends some comfort to accept that individual differences in protected values and Honesty-Humility, as hypothesized, indeed play a significant role in predicting corrupt behaviors in practice.

In a supplementary online appendix (S3 Appendix), we provide analyses on the size of the bribe offered and the minimum size of the bribe accepted by participants. The results show that the size of the bribe offered and accepted is–as already proposed by [10]–indeed a strategic decision (based on the specific parameters of the bribe setting) and hence largely unrelated to protected values, Honesty-Humility, and the control variables.

## Discussion and conclusion

Corruption is a widespread phenomenon and has numerous negative consequences [2]. Extant research has greatly enhanced our understanding of aggregated differences in corruption between countries and organizations. In contrast, our knowledge about why people within the same context seem to differ in their susceptibility to engage in corruption [12, 23] and why we see within-company variations in corrupt behavior [28] is still severely limited. To encourage the fight against corruption, theory-building efforts, as well as practice, thus stand to gain from shedding more light on who is more likely to resist corruption and why. The present research contributes to this gap by empirically studying the role of moral commitment for predicting resistance in corrupt behavior.

The results of our two-part study show that individuals refrain from corruption the more they experience that compromising their integrity for monetary gains is unacceptable and the higher their level of Honesty-Humility. Notably, the results also remain robust to the inclusion of several other personal characteristics that have been suggested to more broadly shape engagement in corrupt or unethical behaviors.

Among the proxies of moral commitment included in this study, PV_RC_ is the most robust predictor of rejecting bribery, while Honesty-Humility appears to be more powerful in predicting Bribe-Offering. One interpretation of this result may be that PV_RC_ reflects identity implications more than the other proxies (PV_NT_, Honesty-Humility) do. Furthermore, accepting a bribe may (more than even offering a bribe) reflect to a person an undesirable identity; that is, it reflects that she is corruptible. This is in line with [86], who found that subtle linguistic changes in the experimental materials affect cheating. Specifically, they found that saying, “please, don’t be a cheater” was more effective in reducing cheating among participants than saying, “please, don’t cheat.” The main difference between these two instructions is that the latter focuses on action, whereas the former focuses on the actor’s identity. An instruction that implicates the self is likely to invoke identity concerns and a desire to maintain a positive self-image. Similarly, for individuals judging bribery as unacceptable, blameworthy, and outrageous, being faced with a bribe is likely to put their image as an integer and incorruptible person at risk when accepting the bribe. This concern may be particularly powerful in activating resistance to corruption.

Our study also adds to previous literature studying the relation between Honesty-Humility and corruption. Previous studies have shown that individuals with higher levels of Honesty-Humility were less likely to cheat in corrupted collaboration settings [15], and less likely to take a bribe [16]. Importantly, our study adds to those previous studies by examining the behavior of both, the bribee and the briber, thereby allowing to compare the explanatory power of intrapersonal factors in resisting Bribe-Offering and/or Bribe-Taking. Interestingly, the results reveals that Honesty-Humility is more powerful in predicting Bribe-Offering than Bribe-Taking. We speculate that, in contrast to PV_RC_, Honesty-Humility is more likely to relate to active (un)ethical behavior. It includes the dimension that individuals do not initiate behavior or bend norms at the expense of others to increase their individual benefit. This is potentially why Honesty-Humility is more important in refraining from initiating a corruptive act (e.g., offering a bribe) than rejecting a bribe in our study.

We believe that our findings are important for research and practice in at least three ways. They extend previous research on corruption by shedding more light on the role of moral commitment in corrupt acts, thereby better accounting for individual differences which have been largely neglected in past corruption research (e.g., [3, 4, 87]). We add to this research by showing that moral commitment is important for understanding heterogeneity in corrupt behavior. Thus, our findings underpin that broadening the research perspective on the antecedents of corruption beyond macro-level factors by including individual-level factors promises to contribute to our understanding of corruption and, ultimately, to developing more nuanced recommendations mitigating corruption in practice. As [28] noted, the notion of moral commitment points out that nonmonetary incentives are likely to matter in fighting corruption. This also extends recent survey-based research among business professionals, suggesting that stable traits are related to whether professionals condone bribery or not [33]. Our results add to this by highlighting the role of stable values in the engagement in bribery.

In addition, we contribute to prior literature by showing that moral commitment matters in settings of interdependent decision-making. Typically, research has examined antecedents of decision making and behavior by framing unethical behavior mainly as an individual decision problem (e.g., cheating or fraud). We add to this stream of literature by showing the pivotal role of moral commitment in a setting that involves cooperation between two parties to enhance their outcome when directly harming a third party.

Finally, our results have implications for organizational practice. Classical approaches to combating corruption problems are based on redefining and strengthening laws, updating codes of conduct, and implementing control and sanctions [88, 89]. Meanwhile, Information and Communication Technologies (ICT) are increasingly used to track individual behavior to deter corruption (e.g., [90, 91]). However, implementing and operating control systems can be costly and may provoke dysfunctional effects (e.g., [92–94]). Beyond these strategies to prevent corruption, our results point to the importance of hiring employees committed to ethical values and, more importantly, appointing such individuals to positions susceptible to corrupt offers (e.g., procurement managers). For instance, hiring and retaining individuals who consider compromising their protected values to be unacceptable (officials high in PV_RC_) can reduce the likelihood that these officers accept bribes and, hence, are a means of fighting corruption. Cleary, measuring who is susceptible to corruption is not easy and poses a challenge in practice. But it seems essential that companies start placing more emphasis on moral values during job recruitment and promotion processes. Furthermore, since many corrupt acts are likely to remain *hidden*, it seems important to complement conventional strategies by designing programs that seek to promote an individual’s moral commitment. One possibility may be to implement ethical nugdes in the surrounding to remind people of moral standards, thereby strengthening the role of moral values in shaping behavior (e.g., [31, 95]).

A limitation of the present study is that the laboratory experiment was conducted with student participants rather than practitioners. Replicating our study using a sample of individuals without a university background and with older individuals would help determine the extent to which our findings generalize to individuals with significantly different backgrounds; these two factors might affect the values that individuals hold and individuals’ willingness to engage in corrupt behavior, respectively. In addition, though conducting a laboratory experiment has some methodological strengths, they can be criticized because of their artificiality. It is open so far whether the findings of our study would also hold in real-life settings (though prior research supports the external validity of laboratory research on corruption, e.g., [96]). Additionally, our experiment has limited statistical power due to its relatively small sample size. We used the full experimental setup including participants in the role of the “other member of society”, who did not make any decision and thus provided no data. Including this group seemed important because this group is affected by the other participants’ behavior (and, importantly, the others knew that they directly affect the remuneration of these participants) and because the other participants might behave differently if the "other member of society” were only a computer playing this role rather than some real person. This limited power is evident when we split-up our full sample to analyze offering or accepting a bribe separately. We conducted a sensitivity power analysis and observe that it would require medium-sized relations between Honesty-Humility/PV_RC_ and Bribe-Accepting/Bribe-Offering (*r* = - 0.31) to obtain 80% power in a two-tailed test with our sample size of 76 participants. However, we observe smaller relations (see Table 2). We also had to dichotomize our indicators of accepting or offering a bribe, which further reduced power. Hence, it would be preferrable for future research to rely on greater sample sizes or use experimental settings where the size of the bribe can be meaningfully interpreted. Nonetheless, given the fact that extensive prior research confirms the important role of protected values and Honesty-Humility in (un)ethical behavior, we are confident that our results also hold in larger samples.

We also intentionally chose a one-shot game to minimize reputation effects, thereby facilitating the distillation of the pure effect of protected values and Honesty-Humility. Although we could show that people high in moral commitment are more steadfast, the question remains as to whether this steadfastness remains over time. Indeed, some people may give up after a specific time and, for example, leave the company [14]. Thus, a promising avenue for future research could be to examine how this resistance may change in multi-round designs. Furthermore, the participants in our experiment who engaged in corrupt behaviors did not risk punishment for their behavior. Corruption in some real-world settings may not imply the risk of punishment, whether financial or reputational. However, further research is necessary to test the role of protected values and Honesty-Humility in situations where there is a credible threat of ex-post punishment of corrupt behaviors. Additionally, it is worth emphasizing that we relied on a quasi-experimental setup to test for the role of moral commitment. To examine the causal influence of moral commitment and to be able to assign people randomly to a high an low commitment condition, future studies may use a design to manipulate moral commitment rather than to measure it in advance. Moreover, whereas we took great care to minimize the risk of experimenter demand effects, another possibility for future studies may be to follow recent recommendations on assessing the presence of experimenter demand effects by comparing results to a control sample for which high experimenter effects are deliberately induced [97, 98].

Also note that in both our main analyses and our robustness tests, the number of semesters in which the participants have been enrolled turned out to be a statistically significant predictor of offering a bribe. Given the various classes covering ethics and stressing (social) responsibility that students take during their study programs, this finding is, at the least, surprising, even more so because the variable Semesters does not seem to heighten the probability that a participant (in the role of a “public official”) accepts a bribe. We do not have a definite explanation for this finding, though one reason could be that more senior students have more experience with economic games and thus are more experienced and willing to maximize their remuneration in laboratory experiments. Future studies might want to control for, in a more fine-grained manner, whether and what courses students took to observe the effect that courses covering ethics and stressing (social) responsibility have on engagement in corrupt behavior or whether or to what degree other courses interfere with or even overcompensate for the effects of courses covering ethics and stressing (social) responsibility. Such studies could inform curriculum building.

We believe that this research points to additional avenues for future research. For example, beyond using protected values and Honesty-Humility as proxies for moral commitment, there are, of course, other candidates of concepts that could be considered as valuable proxies for moral commitment. Future research may compare the role of protected values and Honesty-Humility with other possible proxies of moral commitment, such as the role of guilt-proneness [99] or moral identity [55]. Furthermore, we relied on a subset of potential participant characteristics, but future research could examine, for example, how participants’ individual wealth or their family background might be associated with the likelihood to engage in bribery. Additional studies could explore in greater detail how protected values and Honesty-Humility affect motivational, volitional, affective, and cognitive components of decision making and behavior and how they interact with other contextual features (such as punishment and social norms). Another promising avenue for future research could also be to investigate how people endorsing protected values and Honesty-Humility affect other parties (observing their behavior) and whether such people can contribute to the diffusion of non-corrupt behavior.

In conclusion, the present findings provide novel insights into the role of moral commitment in corruption. They suggest that assessing proxies of moral commitment is helpful to better understand individual differences in corrupt behavior, and taking account of such factors in practice (e.g., for staff selection) may likely assist in deterring corruption.

## Supporting information

S1 Appendix Items and experimental instructions. (DOCX)

S2 Appendix Flow chart of study and corruption paradigm. (TIF)

S3 Appendix Additional analyses. (DOCX)
