# Supplementary material for: Does moral commitment predict resistance to corruption? experimental evidence from a bribery game
Source: PLoS One. 2022 Jan 11;17(1):e0262201. doi: 10.1371/journal.pone.0262201 (PMC8752004; doi:10.1371/journal.pone.0262201)
Supplement: S1 Appendix — (DOCX) [file pone.0262201.s001.docx]

**S1 Appendix. Items and Experimental Instructions**

**A Scales and Items included in the Online Survey Part**

*1. Measure of Honesty-Humility* (after Ashton & Lee, 2009)

On the following pages, you can find statements that may apply more or less to yourself.

Please indicate how much you (dis)agree or disagree with each statement*.* (1 = *strongly disagree*, 7 = *strongly agree*)

- I wouldn’t use flattery to get a raise or promotion at work, even if I thought it would succeed.
- If I want something from someone, I will laugh at that person’s worst jokes. (R)
- I wouldn’t pretend to like someone just to get that person to do favors for me.
- If I knew that I could never get caught, I would be willing to steal a million dollars. (R)
- I would never accept a bribe, even if it were very large.
- I’d be tempted to use counterfeit money, if I were sure I could get away with it. (R)
- Having a lot of money is not especially important to me.
- I would get a lot of pleasure from owning expensive luxury goods. (R)
- I think that I am entitled to more respect than the average person is. (R)
- I want people to know that I am an important person of high status. (R)

*2. Measure of Dispositional Greed* (after Krekels & Pandelaere, 2015)

Please indicate how much you (dis)agree with each statement (1 = *strongly disagree*, 7 = *strongly agree*)

- No matter how much I have of something, I always want more.
- One can never have enough.
- Even when I am fulfilled, I often seek more.
- The pursuit of more and better is an important goal in life for me.
- A simple basic life is sufficient for me. (R)
- I am easily satisfied with what I’ve got.(R)

*3. Measure of Protected Values_RC_* (= reactions to compromising PV) (after Tanner et al., 2009)

Situation 1: Consider a patient offering his/her doctor an invitation to a fine dining restaurant to get an appointment earlier than another patient and the doctor accepting the invitation.

What do you think about people proposing such an offer (such as e.g. the patient)?

- -2 *= not at all praiseworthy,* +2 *= very much praiseworthy (R)*
- -2 *= not at all blameworthy,* +2 *= very much blameworthy*
- -2 *= not at all outrageous,* +2 *= very much outrageous*
- -2 *= not at all acceptable,* +2 *= very much acceptable (R)*

What do you think about people accepting such an offer (such as e.g. the doctor)?

- -2 *= not at all praiseworthy,* +2 *= very much praiseworthy (R)*
- -2 *= not at all blameworthy,* +2 *= very much blameworthy*
- -2 *= not at all outrageous,* +2 *= very much outrageous*
- -2 *= not at all acceptable,* +2 *= very much acceptable (R)*

*4. Measure of Protected Values_RC_* (= reactions to compromising PV) (after Tanner et al., 2009)

Situation 2: Consider a company manager offering the local municipality’s Department of Planning & Building money to get the permission to build a new factory in a nature reserve. The head of the local Department of Planning & Building accepts the offer.

What do you think about people proposing such an offer (such as e.g. the company manager)?

- -2 *= not at all praiseworthy,* +2 *= very much praiseworthy (R)*
- -2 *= not at all blameworthy,* +2 *= very much blameworthy*
- -2 *= not at all outrageous,* +2 *= very much outrageous*
- -2 *= not at all acceptable,* +2 *= very much acceptable (R)*

What do you think about people accepting such an offer (such as e.g. the head of the local Department of Planning & Building)?

- -2 *= not at all praiseworthy,* +2 *= very much praiseworthy (R)*
- -2 *= not at all blameworthy,* +2 *= very much blameworthy*
- -2 *= not at all outrageous,* +2 *= very much outrageous*
- -2 *= not at all acceptable,* +2 *= very much acceptable (R)*

*5. Measure of Protected Values_NT_* (= no trade-offs with PV) (after Tanner et al., 2009)

There are situations where two individuals can engage in an arrangement that has positive consequences for these two individuals, but negative consequences for an unrelated third individual. Some view such arrangements as a violent of integrity; others regard it as acceptable protection of personal interests.

To what extent do you agree with the following statements? (1 = *strongly disagree*, 7 = *strongly agree*)

Integrity is something…

… that one should not sacrifice, no matter what the (material or other) benefits are.

… for which it is right to make a cost-benefit analysis. (R)

… that cannot be measured in monetary terms.

… about which one can be flexible if the situation demands it. (R)

*6. Measure of Community Commitment* (after Burroughs & Rindfleisch, 2002)

Please indicate how much you agree to the following statements*.* (1 = *strongly disagree*, 7 = *strongly agree*)

- I believe that the more money you have, the happier you are.
- I value money very highly.
- I feel an obligation to donate money to local charities.
- I feel that it is important to serve as a volunteer in my community.
- It is important to me to form close ties with others in my community.
- I am very concerned about the welfare of my community.
- I believe it is important to take an active role in the civic affairs of the community in which I live.
- I believe it is important to attend town hall or city council meetings and voice one’s concerns about issues affecting the community.

*7. Measure of Cultural Identification*

Do you identify culturally with a country other than (or in addition to) France?

- No
- Yes, I do.
  - Which other country than France or in addition to France do you identify culturally with?
    Select ______________

*8. Measure of Risk Tolerance* (after Dohmen et al., 2011)

How do you see yourself? Are you generally a person who is fully prepared to take risks or do you try to avoid taking risks?

- I am generally a person who is…(1 = *not at all willing to take risks*; 10 = *very willing to take risks*)

*9. Measure of Trait Competitiveness* (after Brown et al., 1998)

Please indicate how much you (dis)agree with each statement. (1 = *strongly disagree*, 7 = *strongly agree*)

- I enjoy working in situations involving competition with others.
- It is important to me to perform better than others on a task.
- I feel that winning is important in both work and games.
- I try harder when I am in competition with other people.

*10. Measure of Religious Strength*

- How religious would you describe yourself? (1 = *not at all religious*, 5 = *very religious*; *no answer*)
- How important is your religion / your faith to you? (1 = *not at all important*, 5 = *very important*; *no answer*)

**B Instructions of the Laboratory Experiment Part**

***Instructions***

*The game you are going to pay involves 15 players, that is: 5 Private Citizens, 5 Public Officials, and 5 Other Members of Society. Each one of you will play this game in the role of either Private Citizen, Public Official or Other Member of society, with 14 other students. Each Private Citizen is randomly matched with a Public Official. However, no one will know exactly who is playing with whom.*

*Each one of you will receive an initial endowment of “cash” denoted in “gilpets” (the currency we use here in this game). Each Private Citizen will get 35 gilpets; each Public Official 35 gilpets; each Other Member of Society 25 gilpets.*

*The game proceeds as follows:*

*First, every Private Citizen can offer a bribe to the Public Official with whom he/she is playing. He/she can decide to offer either nothing or any amount between 1 gilpet and 20 gilpets.
Second, every Public Official has to decide whether or not to accept the bribe offered by the Private Citizen with whom he/she is matched. If the Private Citizen offers a bribe and the Public Official rejects the offer, then the Public Official gets 35 gilpets, wheras the Private Citizens get 34 gilpets. The payoffs of the Other Members of Society are not affected. If the Private Citizen decides to offer a bribe and the Public Official decides to accept the offer, then the payoffs for the Private Citizen and the Public Official can be found in the Table 1, that you find on your desk.*

*On the next two pages we will walk you through an example and explain to you how the payoffs for the Other Member of Society look like. Please click on “Continue”.*

*------------------------------------*

***Example***

*Look at* ***Table 1*** *on your desk that provides all the information about how your and the other participants choices affect your payoff. It is designed to help you to decide how to play the game.*

*The first column on the left of the table shows you all the possible bribes that a Private Citizen could offer to a Public Official. So, in the top row the Private Citizen is offering zero gilpet to the Public Official, i.e. no bribe, and in the bottom row the Private Citizen is offering a bribe equal to 20 gilpets to the Public Official. All the other possible bribes are listed in between.*

*To the right of this first column, you have two sets of three columns.*

*The first set of three shows you what happens to the payoffs of the Private Citizen, the Public Official and each Other Member of Society if the Public Official decides* ***to accept the offer*** *offered by the Private Citizen.*

*The second set of three shows you what happens to the payoffs of the Private Citizen, the Public Official and each Other Member of Society, if the Public Official decides* ***to reject the offer*** *offered by the Private Citizen.*

*It is important that you understand the table on your desk. We will walk you through an example:*

*Suppose the Private Citizen offers a bribe equal to 12 gilpets to the Public Official. Then if the Public Official chooses to accept the offer, the Private Citizen will go home with 38 gilpets, the Public Official will go home with 42 gilpets, and each Other Member of Society will suffer a loss of 4 gilpet. If, instead, the Public Official decides to reject the offer, the Private Citizen goes home with 34 gilpet, the Public Official goes home with 35 gilpets, and each Other Member of Society does not suffer a monetary loss, that is: s/he goes home with the initial endowment of 25 gilpets.*

*So each Private Citizen has to decide whether or not to offer a bribe (and the amount of the bribe, if any) and each Public Official has to decide whether or not accept the bribe offered.*

***Table 1***

|  | |  | ***Public official decides to…*** | | | | |
| --- | --- | --- | --- | --- | --- | --- | --- |
|  |  |  | ***Accept*** | | ***Reject*** | | |
| ***Private citizen decides to offer a bribe equal to…*** |  | ***Private citizen*** | ***Public official*** | ***Each Other Member of Society*** | ***Private citizen*** | ***Public official*** | ***Each Other Member of Society*** |
|  | ***0*** | *35* | *35* | *unaffected* | *35* | *35* | *unaffected* |
|  | ***1*** | *49* | *31* | *suffers a loss of 4G* | *34* | *35* | *unaffected* |
|  | ***2*** | *48* | *32* | *suffers a loss of 4G* | *34* | *35* | *unaffected* |
|  | ***3*** | *47* | *33* | *suffers a loss of 4G* | *34* | *35* | *unaffected* |
|  | ***4*** | *46* | *34* | *suffers a loss of 4G* | *34* | *35* | *unaffected* |
|  | ***5*** | *45* | *35* | *suffers a loss of 4G* | *34* | *35* | *unaffected* |
|  | ***6*** | *44* | *36* | *suffers a loss of 4G* | *34* | *35* | *unaffected* |
|  | ***7*** | *43* | *37* | *suffers a loss of 4G* | *34* | *35* | *unaffected* |
|  | ***8*** | *42* | *38* | *suffers a loss of 4G* | *34* | *35* | *unaffected* |
|  | ***9*** | *41* | *39* | *suffers a loss of 4G* | *34* | *35* | *unaffected* |
|  | ***10*** | *40* | *40* | *suffers a loss of 4G* | *34* | *35* | *unaffected* |
|  | ***11*** | *39* | *41* | *suffers a loss of 4G* | *34* | *35* | *unaffected* |
|  | ***12*** | *38* | *42* | *suffers a loss of 4G* | *34* | *35* | *unaffected* |
|  | ***13*** | *37* | *43* | *suffers a loss of 4G* | *34* | *35* | *unaffected* |
|  | ***14*** | *36* | *44* | *suffers a loss of 4G* | *34* | *35* | *unaffected* |
|  | ***15*** | *35* | *45* | *suffers a loss of 4G* | *34* | *35* | *unaffected* |
|  | ***16*** | *34* | *46* | *suffers a loss of 4G* | *34* | *35* | *unaffected* |
|  | ***17*** | *33* | *47* | *suffers a loss of 4G* | *34* | *35* | *unaffected* |
|  | ***18*** | *32* | *48* | *suffers a loss of 4G* | *34* | *35* | *unaffected* |
|  | ***19*** | *31* | *49* | *suffers a loss of 4G* | *34* | *35* | *unaffected* |
|  | ***20*** | *30* | *50* | *suffers a loss of 4G* | *34* | *35* | *unaffected* |

*------------------------------------*

***Example (Continued)***

*Let’s now look at the payoffs of each Other Member of Society.*

*Please look at the Table 2 on your desk.*

*The first row of the table indicates the payoffs of each and every Other Member of Society when no bribe has been and accepted.*

*The second row indicates the payoffs when 1 bribe has been offered by a Private Citizen and has been accepted by matched Public Official.*

*The third row indicates the payoffs of each and every Other Member of Society when 2 bribes have been offered and accepted, and son an and so forth.*

*Therefore each Other Member of Society can earn from the game a maximum of 35 gilpets, equal to their initial endowment, if no bribe is offered and accepted, and a minimum of 5 gilpets if all Private Citizen offer a bribe and all Public Officials decide to accept the offered bribes.*

*So each Other Member of Society does not make any decisions in this game, but she/he suffers a loss of 4 gilpets for any bribe that is offered by a Private Citizen and accepted by the matched Public Official. If all the Private Citizens decide to offer a bribe and all the Public Officials decide to accept the offered bribe, then each Other Member of Society suffers a total loss of 20 gilpets.*

***Table 2***

| ***Each Other Members of Society takes home …*** | ***Payoff*** |
| --- | --- |
| *If no bribe is offered and accepted (or ribe is offered, but rejected by matched Official)* | *25* |
| *If one Citizen decides to offer a bribe and the bribe is accepted by the matched Official* | *21* |
| *If two Citizens decide to offer a bribe and the bribes are accepted by the matched Officials* | *17* |
| *If three Citizens decide to offer a bribe and the bribes are accepted by the matched Officials* | *13* |
| *If four Citizens decide to offer a bribe and the bribes are accepted by the matched Officials* | *9* |
| *If five Citizens decide to offer a bribe and the bribes are accepted by the matched Officials* | *5* |

*------------------------------------*

***Test questions (1/7)***

*In the following, we introduce some test questions to make sure that you understand how yours, ad the other participants’ choice affect the payoffs.*

*Suppose the Private Citizen offers a bribe equal to* ***8 gilpets*** *to the Public Official. Then if the Public Official* ***choose to accept*** *the offer.*

| *How much does the* ***Private Citizen*** *get?* | - *34 gilpets* - *42 gilpets* - *47 gilpets* |
| --- | --- |

*------------------------------------*

***Test questions (2/7)***

*Suppose the Private Citizen offers a bribe equal to* ***8 gilpets*** *to the Public Official. Then if the Public Official* ***choose to accept*** *the offer.*

| *How much does the* ***Public Official*** *get?* | - *27 gilpets* - *33 gilpets* - *38 gilpets* |
| --- | --- |

*------------------------------------*

***Test questions (3/7)***

*Suppose the Private Citizen offers a bribe equal to* ***8 gilpets*** *to the Public Official. Then if the Public Official* ***choose to accept*** *the offer.*

*How does this bribe affect each* ***Other Member of Society****?*

- *each Other Member of Society will suffer a loss of 4 gilpets*
- *each Other Member of Society will suffer a loss of 8 gilpets*
- *each Other Member of Society will suffer a loss of 2 gilpets*

*------------------------------------*

***Test questions (4/7)***

*Suppose the Private Citizen offers a bribe equal to* ***8 gilpets*** *to the Public Official. Then if the Public Official* ***choose to reject*** *the offer.*

| *How much does the* ***Private Citizen*** *get?* | - *31 gilpets* - *34 gilpets* - *37 gilpets* |
| --- | --- |

*------------------------------------*

***Test questions (5/7)***

*Suppose the Private Citizen offers a bribe equal to* ***8 gilpets*** *to the Public Official. Then if the Public Official* ***choose to reject*** *the offer.*

| *How much does the* ***Public Official*** *get?* | - *32 gilpets* - *35 gilpets* - *38 gilpets* |
| --- | --- |

*------------------------------------*

***Test questions (6/7)***

*Suppose the Private Citizen offers a bribe equal to* ***8 gilpets*** *to the Public Official. Then if the Public Official* ***choose to reject*** *the offer.*

*How does this bribe affect each* ***Other Member of Society****?*

- *each Other Member of Society will suffer a loss of 4 gilpets*
- *each Other Member of Society will suffer a loss of 8 gilpets*
- *each Other Member of Society will not suffer a monetary loss (i.e. his/her payoffs are unaffected)*

*------------------------------------*

***Test questions (7/7)***

*Suppose that* ***3*** *Private Citizens in this room* ***successfully*** *offer a bribe to their Public Official (=the Public Official accepted the offer)*

*How do these three successful bribes affect each* ***Other Member of Society****?*

- *each Other Member of Society will suffer a loss of 4 gilpets and, hence, goes home with 21 gilpets*
- *each Other Member of Society will suffer a loss of 12 gilpets and, hence, goes home with 13 gilpets*
- *each Other Member of Society will suffer a loss of 20 gilpets and, hence, goes home with 5 gilpets*

*------------------------------------*

***Decision***

***You are assigned the role of a Private Citizen!***

*You now have the opportunity to offer a bribe or chose not to offer a bribe. If you wish to offer any bribe between 1 and20 gilpets to a Public Official, please select “Yes” and a box will appear to indicate the amount.*

| *I wish to offer a bribe* | - *Yes* - *No* |
| --- | --- |

*------------------------------------*

***Decision***

***You are assigned the role of a Public Official!***

*Public Officials, on the following page, you will be given a Response Form.*

*Question 1 asks what you would do if the Private Citizen offers you a bribe equal to 1 gilpet. Would you accept so that you get 31 gilpets, the Private Citizen gets 49 gilpets and each and every Other Member of Society suffers a loss of 4? Or would you reject so that you get 35 gilpets, the Private Citizen gets 34 gilpets and each and every Other Member of Society does not suffer a loss of 4? You have to tick the box next to the option you choose.*

*Question 2 asks what you would do if Private Citizen sends you a bribe equal to 2 gilpets and so on and so forth, until the last question which asks you what you would do if the Private Citizen sends you 20 gilpets.*

*One of the responses you put on this from will determine your payoffs, so think carefully when completing the forms. Later the computer will match the form filled in by the Public Officials with the bribe offered by the Private Citizens and do as the Officials have indicated on the form.*

*------------------------------------*

***Decision (1/2)***

*If the Private Citizen sends you…*

*… a bribe equal to 1 gilpet, what would you do?*

- *Accept: so that you get 31, the Private Citizen gets 49 and each and every Other Member of Society suffers a loss of 4?*
- *Reject: so that you get 35, the Private Citizen gets 34 and each and every Other Member of Society does not suffer a loss of 4?*

*… a bribe equal to 2 gilpets, what would you do?*

- *Accept: so that you get 32, the Private Citizen gets 48 and each and every Other Member of Society suffers a loss of 4?*
- *Reject: so that you get 35, the Private Citizen gets 34 and each and every Other Member of Society does not suffer a loss of 4?*

*… a bribe equal to 3 gilpets, what would you do?*

- *Accept: so that you get 33, the Private Citizen gets 47 and each and every Other Member of Society suffers a loss of 4?*
- *Reject: so that you get 35, the Private Citizen gets 34 and each and every Other Member of Society does not suffer a loss of 4?*

*… a bribe equal to 4 gilpets, what would you do?*

- *Accept: so that you get 34, the Private Citizen gets 46 and each and every Other Member of Society suffers a loss of 4?*
- *Reject: so that you get 35, the Private Citizen gets 34 and each and every Other Member of Society does not suffer a loss of 4?*

*… a bribe equal to 5 gilpets, what would you do?*

- *Accept: so that you get 35, the Private Citizen gets 45 and each and every Other Member of Society suffers a loss of 4?*
- *Reject: so that you get 35, the Private Citizen gets 34 and each and every Other Member of Society does not suffer a loss of 4?*

*… a bribe equal to 6 gilpets, what would you do?*

- *Accept: so that you get 36, the Private Citizen gets 44 and each and every Other Member of Society suffers a loss of 4?*
- *Reject: so that you get 35, the Private Citizen gets 34 and each and every Other Member of Society does not suffer a loss of 4?*

*… a bribe equal to 7 gilpets, what would you do?*

- *Accept: so that you get 37, the Private Citizen gets 43 and each and every Other Member of Society suffers a loss of 4?*
- *Reject: so that you get 35, the Private Citizen gets 34 and each and every Other Member of Society does not suffer a loss of 4?*

*… a bribe equal to 8 gilpets, what would you do?*

- *Accept: so that you get 38, the Private Citizen gets 42 and each and every Other Member of Society suffers a loss of 4?*
- *Reject: so that you get 35, the Private Citizen gets 34 and each and every Other Member of Society does not suffer a loss of 4?*

*… a bribe equal to 9 gilpets, what would you do?*

- *Accept: so that you get 39, the Private Citizen gets 41 and each and every Other Member of Society suffers a loss of 4?*
- *Reject: so that you get 35, the Private Citizen gets 34 and each and every Other Member of Society does not suffer a loss of 4?*

*… a bribe equal to 10 gilpets, what would you do?*

- *Accept: so that you get 40, the Private Citizen gets 40 and each and every Other Member of Society suffers a loss of 4?*
- *Reject: so that you get 35, the Private Citizen gets 34 and each and every Other Member of Society does not suffer a loss of 4?*

*------------------------------------*

***Decision (2/2)***

*If the Private Citizen sends you…*

*… a bribe equal to 11 gilpets, what would you do?*

- *Accept: so that you get 41, the Private Citizen gets 39 and each and every Other Member of Society suffers a loss of 4?*
- *Reject: so that you get 35, the Private Citizen gets 34 and each and every Other Member of Society does not suffer a loss of 4?*

*… a bribe equal to 12 gilpets, what would you do?*

- *Accept: so that you get 42, the Private Citizen gets 38 and each and every Other Member of Society suffers a loss of 4?*
- *Reject: so that you get 35, the Private Citizen gets 34 and each and every Other Member of Society does not suffer a loss of 4?*

*… a bribe equal to 13 gilpets, what would you do?*

- *Accept: so that you get 43, the Private Citizen gets 37 and each and every Other Member of Society suffers a loss of 4?*
- *Reject: so that you get 35, the Private Citizen gets 34 and each and every Other Member of Society does not suffer a loss of 4?*

*… a bribe equal to 14 gilpets, what would you do?*

- *Accept: so that you get 44, the Private Citizen gets 36 and each and every Other Member of Society suffers a loss of 4?*
- *Reject: so that you get 35, the Private Citizen gets 34 and each and every Other Member of Society does not suffer a loss of 4?*

*… a bribe equal to 15 gilpets, what would you do?*

- *Accept: so that you get 45, the Private Citizen gets 35 and each and every Other Member of Society suffers a loss of 4?*
- *Reject: so that you get 35, the Private Citizen gets 34 and each and every Other Member of Society does not suffer a loss of 4?*

*… a bribe equal to 16 gilpets, what would you do?*

- *Accept: so that you get 46, the Private Citizen gets 34 and each and every Other Member of Society suffers a loss of 4?*
- *Reject: so that you get 35, the Private Citizen gets 34 and each and every Other Member of Society does not suffer a loss of 4?*

*… a bribe equal to 17 gilpets, what would you do?*

- *Accept: so that you get 47, the Private Citizen gets 33 and each and every Other Member of Society suffers a loss of 4?*
- *Reject: so that you get 35, the Private Citizen gets 34 and each and every Other Member of Society does not suffer a loss of 4?*

*… a bribe equal to 18 gilpets, what would you do?*

- *Accept: so that you get 48, the Private Citizen gets 32 and each and every Other Member of Society suffers a loss of 4?*
- *Reject: so that you get 35, the Private Citizen gets 34 and each and every Other Member of Society does not suffer a loss of 4?*

*… a bribe equal to 19 gilpets, what would you do?*

- *Accept: so that you get 49, the Private Citizen gets 31 and each and every Other Member of Society suffers a loss of 4?*
- *Reject: so that you get 35, the Private Citizen gets 34 and each and every Other Member of Society does not suffer a loss of 4?*

*… a bribe equal to 20 gilpets, what would you do?*

- *Accept: so that you get 50, the Private Citizen gets 30 and each and every Other Member of Society suffers a loss of 4?*
- *Reject: so that you get 35, the Private Citizen gets 34 and each and every Other Member of Society does not suffer a loss of 4?*

***Decision***

***You are assigned the role of Other Member of Society!***

*Other Members of Society, you will not make any decision in this game, but your earnings may be reduced, depending on the decisions made by the Citizen and the Official with whom you are matched by the computer. As per Table 2, your payoffs will be reduced if the Private Citizen offers a bribe which is accepted by the Public Official. If more than one bribe is successful, then the payoffs are as in Table 2. Please click on continue and wait until the participants in the role of a Public Official and Pricate Citizen made their choices.*

*------------------------------------*

*You have made your decision and we will ask you to briefly reply to a questionnaire.*

*While you fill in the questionnaire the computer will calculate your remuneration. It will randomly match the choices by the Private Citizen and the choices filled in by the Public Officials. It will do the calculations in gilpets and then convert into EUR. The staff will then prepare your payment and you will receive your payment once you have completed the questionnaire.*

*------------------------------------*

***Your final profit is XXX Euros***

*Please remain seated until the experimenter calls you to settle your compensation. When you are called, please bring along all handouts you received.*
