# Supplementary figures and images for: Does moral commitment predict resistance to corruption? experimental evidence from a bribery game
Source: PLoS One. 2022 Jan 11;17(1):e0262201. doi: 10.1371/journal.pone.0262201 (PMC8752004; doi:10.1371/journal.pone.0262201)

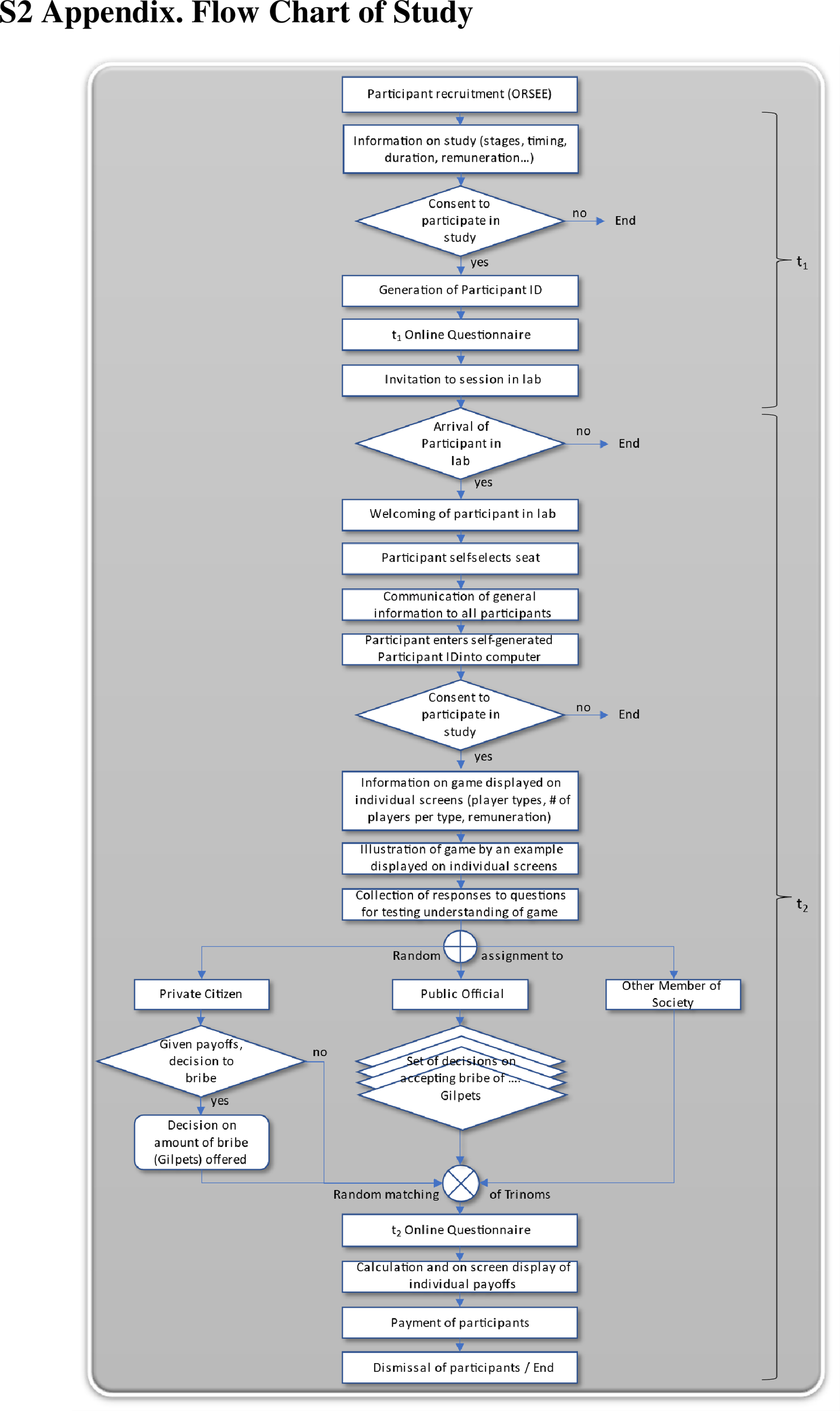

Supplement: S2 Appendix — (TIF) [file pone.0262201.s002.tif]
