# Supplementary material for: Does moral commitment predict resistance to corruption? experimental evidence from a bribery game
Source: PLoS One. 2022 Jan 11;17(1):e0262201. doi: 10.1371/journal.pone.0262201 (PMC8752004; doi:10.1371/journal.pone.0262201)
Supplement: S3 Appendix — (DOCX) [file pone.0262201.s003.docx]

**S3 Appendix. Additional Analyses**

Participants in the “private citizen” role made a two-step decision. First, they were asked whether they want to offer a bribe. If the participants agreed, they were asked to specify the size of the bribe. In the main analyses, we focus on participant’s decision to either engage in or abstain from corrupt behavior, because the size of the bribe is primarily a strategic decision. Descriptive statistics on the size of the bribe offered and the minimum amount accepted are depicted in Figure 2. Figure 2 Panel A plots the bribes offered by participants in the “private citizen” role; Figure 2 Panel B plots the minimum amount that participants in the “public official” role demand to accept a bribe by the “private citizen”.

Figure 1. Amount of Bribe Offered and Minimum Bribe Accepted

Panel A. Bribe Offered


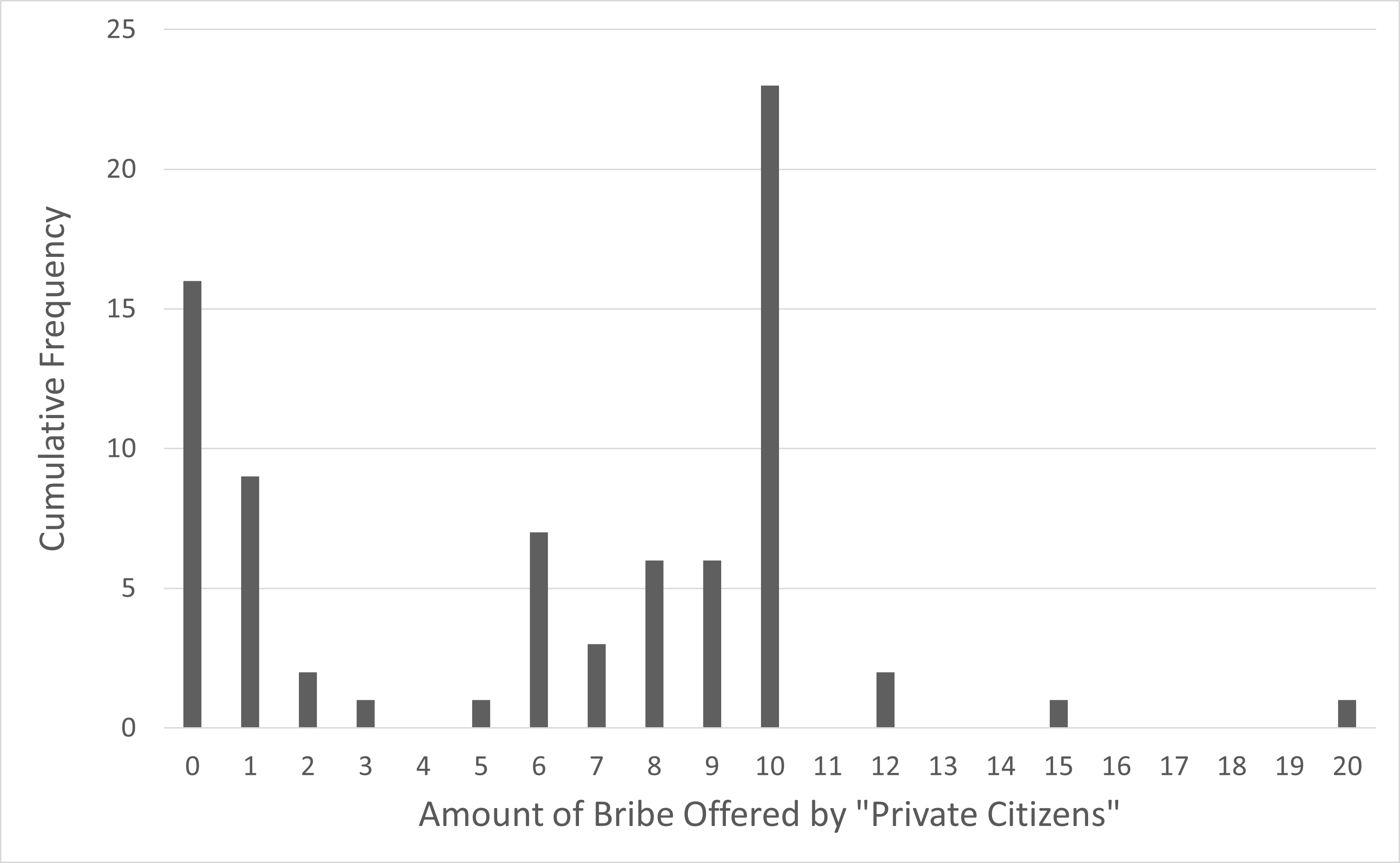


Panel B. Minimum Bribe Accepted


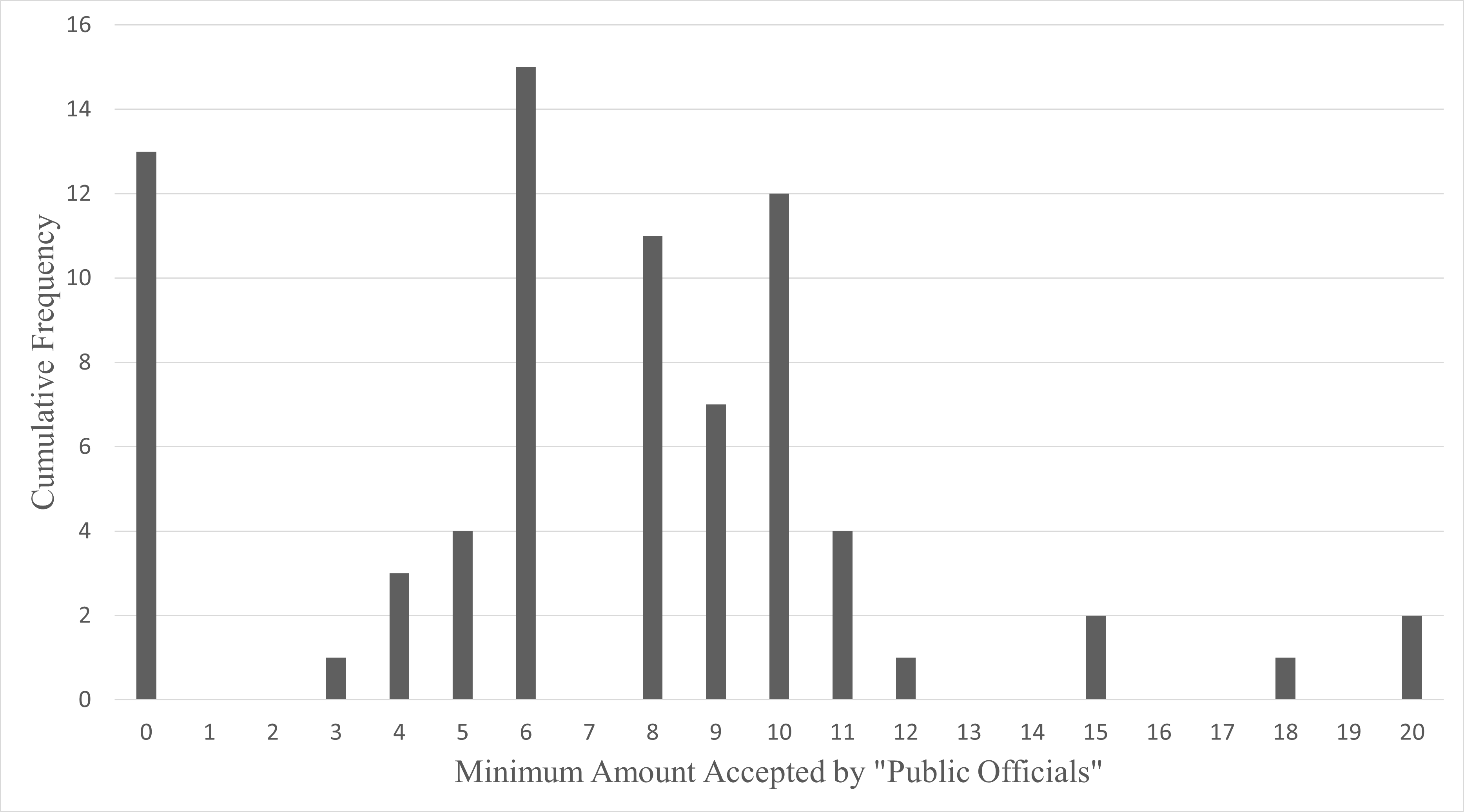


As can be gleaned from the figures, 16 “private citizens” did not offer a bribe and 13 “public officials” did not accept any offer (both have the value zero), showing absolute resistance to engaging in corrupt behavior for financial incentives. Among those participants offering a bribe, most individuals in the role of the “private citizen” (*N* = 23) offer 10 G, which resembles a profit-sharing strategy between the briber and the bribee. Only few participants (*N* = 7) transfer the amount of K + p (namely 5 + 1 = 6 G) to the “public official. Sharing the profit is in fact a good strategy for participants in the role of “private citizens”, when looking at the descriptive results of the minimum amount of a bribe accepted by the “public officials”. Though the relative majority of participants (*N* = 15) accept bribes of at least 6 G (the rational minimum choice of a self-maximizing individual), many “public officials” only accept bribes higher than 8 G(*N* = 11) or 10 G(*N* = 12), i.e. they want the profit to be more fairly shared with the “private citizens” in order to engage in a corrupt action. These descriptive results clearly speak to the fact that the size of the bribe is a strategic decision based on the parameters of the experimental setting.

In line with [14], we do not have a compelling assumption on how personal characteristics or values such as moral commitment are associated with such a strategic bribe size decision. We nevertheless calculate linear regressions with the size of the bribe and the minimum amount accepted as dependent variables and PV_NT_, PV_RC_, Honesty-Humility as independent variables (and control for additional variables in line with our main regression models). Table S2-1 shows the results for the “private citizens” (column 1) with the Bribe Size Offered as the dependent variable and the “public officials” (column 2) with the Minimum Bribe Size Accepted as the dependent variable. The results show that neither of the protected value subscales nor Honesty-Humility are related to the size of the bribe offered or the minimum size of the bribe accepted. Dispositional Greed reduces the amount of the bribe offered and Semesters increases the amount offered. The minimum amount accepted by “public officials” is not significantly related with any of the demographics or individual values measured in the experiment.

**Table S2-1. Robustness Analyses of the Size of the Bribe Offered and Accepted**

|  | (1) | (2) |
| --- | --- | --- |
| VARIABLES | Bribe Size Offered | Minimum Bribe Size Accepted |
|  |  |  |
| PVrc | 0.286 | -0.387 |
|  | (0.838) | (0.839) |
| PVnt | -0.397 | -0.539 |
|  | (0.396) | (0.760) |
| Honesty-Humility | -0.324 | 0.081 |
|  | (0.604) | (0.874) |
| Disp. Greed | -1.129* | 0.259 |
|  | (0.579) | (0.591) |
| Trait Competitiveness | -0.021 | -0.111 |
|  | (0.431) | (0.571) |
| Com. Commitment | -0.356 | 0.422 |
|  | (0.481) | (0.462) |
| Risk Tolerance | 0.298 | -0.198 |
|  | (0.285) | (0.323) |
| CPI | -0.408 | -1.284 |
|  | (1.172) | (0.847) |
| Age | -0.079 | 0.281 |
|  | (0.246) | (0.285) |
| Gender | -0.451 | -0.524 |
|  | (0.729) | (0.934) |
| Semesters | 1.011** | -0.456 |
|  | (0.385) | (0.389) |
| Religiosity | -0.454 | 0.695 |
|  | (0.626) | (0.598) |
|  |  |  |
| Observations | 76 | 76 |
| R-squared | 0.168 | 0.107 |

*Note.* Bribe Sized Offered is the amount that participants in the “private citizen” role offer to the “public official” (column 1). Minimum Bribe Size Accepted is the minimum amount that a participant in the “public official” role demands to accept a bribe (column 2). Gender is dummy codes as 1= *female*, 0 = *male*. CPI is coded as 1 = *low perceived corruption*, 2 = *medium perceived corruption*, 3 = *high perceived corruption*. Robust standard errors are reported in parentheses. * *p* < .10, ** *p* < .05, *** *p* < .01.
